# Supplementary material for: Astrocyte interferon-gamma signaling dampens inflammation during chronic central nervous system autoimmunity via PD-L1
Source: J Neuroinflammation. 2023 Oct 12;20:234. doi: 10.1186/s12974-023-02917-4 (PMC10568873; doi:10.1186/s12974-023-02917-4)
Supplement: Supplementary file 1 — Additional file 1: Figure S1. Expression of key checkpoints in astrocytes stimulated with IFNγ. Primary human spinal cord astrocytes were stimulated with (A) 0, 1, 10, or 100 ng/ml IFNγ for 24 h and RNA was collected and analyzed for transcript levels of CD274. (B) Following stimulation with 10 ng/ml IFNγ, human spinal cord astrocytes were assessed for transcript levels of CD80, CD86, LAG3, CTLA4, TIM3, and TIGIT by qRT PCR. (C) EAE was induced in Ifngr1fl/fl Aldh1l1-CreERT2+ mice (n = 5) and Ifngr1fl/fl littermate controls (n = 3). On day 16 ± 1 mice were injected i.p. with tamoxifen for 5 consecutive days to induce recombination. 35 days post-immunization, mice were perfused, and spinal cords were removed and processed for flow cytometry. Astrocytes were labeled with ACSA-2 to mark astrocytes and PD-1. Cells were gated for singlets, live cells, ACSA-2 positivity, and then PD-1 positive cells. Gating was determined using full minus one (FMO) controls. The number of PD-1+ astrocytes for both genotypes during EAE was quantified. Figure S2. PD-1 agonism and PD-1/PD-L1 antagonism primarily impacts LNCs cocultured with astrocytes. Murine astrocytes and LNCs were harvested and co-cultured in the presence of media alone, 10 ng/ml IFNγ, 100 nM PD-1/PD-L1 inhibitor, and/or 1.0 μg/ml PD-1 agonist for 48 h. Caspase 3/7 activity was measured in (A) LNCs cultured alone, (B) astrocytes co-cultured with LNCs following LNC removal, (C) astrocytes cultured alone, (D) and in a LNC cultured plate following LNC removal to serve as a blank/background control. Caspase 3/7 activity was normalized to cell number. (E) Primary murine LNCs were stimulated with and without 10 ng/ml IFNγ for 24 h and RNA transcript levels of Cd274 and Pdcd1 were assessed. Data are representative of 2 independent experiments with 3–4 technical replicates each. All data represent the mean ± SEM. *P < 0.05 by one-way ANOVA. (F) Following culture with astrocytes, LNCs were processed for flow cytometry and gated fo [file 12974_2023_2917_MOESM1_ESM.pdf]

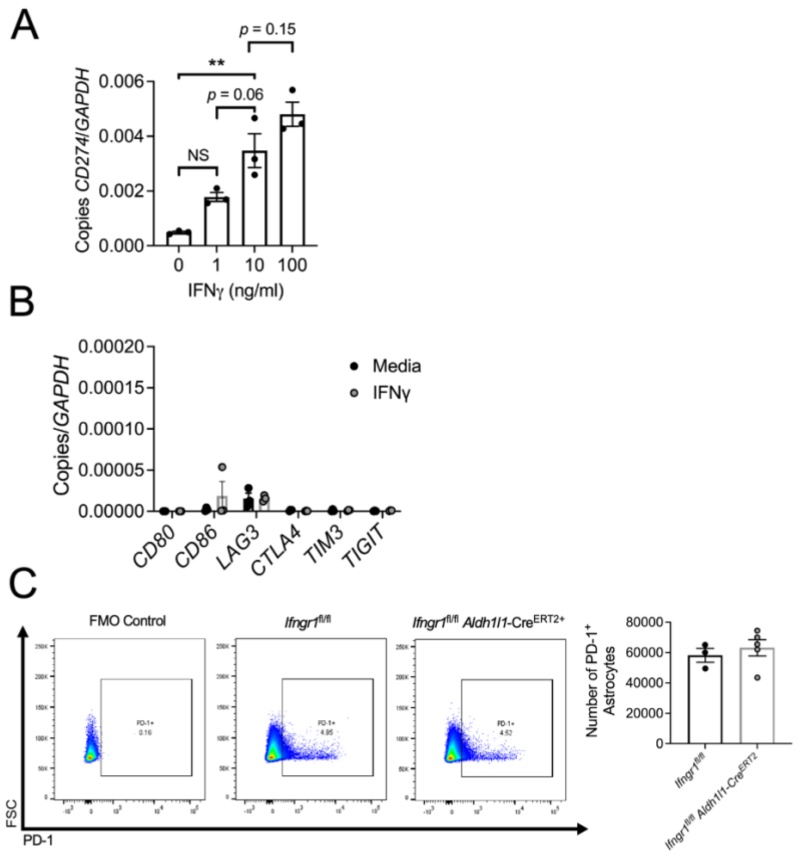

Supplemental Figure 1. Expression of key checkpoints in astrocytes stimulated with IFNγ. Primary human spinal cord astrocytes were stimulated with (**A**) 0, 1, 10, or 100 ng/ml IFNγ for 24 h and RNA was collected and analyzed for transcript levels of *CD274*. (**B**) Following stimulation with 10 ng/ml IFNγ, human spinal cord astrocytes were assessed for transcript levels of *CD80, CD86, LAG3, CTLA4, TIM3,* and *TIGIT* by qRT PCR. (**C**) EAE was induced in *Ifngr1*$^{fl/fl}$ *Aldh1l1*-Cre^{ERT2+} mice (n = 5) and *Ifngr1*$^{fl/fl}$ littermate controls (n = 3). On day 16 ± 1 mice were injected i.p. with tamoxifen for 5 consecutive days to induce recombination. 35 days post-immunization, mice were perfused, and spinal cords were removed and processed for flow cytometry. Astrocytes were labeled with ACSA-2 to mark astrocytes and PD-1. Cells were gated for singlets, live cells, ACSA-2 positivity, and then PD-1 positive cells. Gating was determined using full minus one (FMO) controls. The number of PD-1^{+} astrocytes for both genotypes during EAE was quantified.

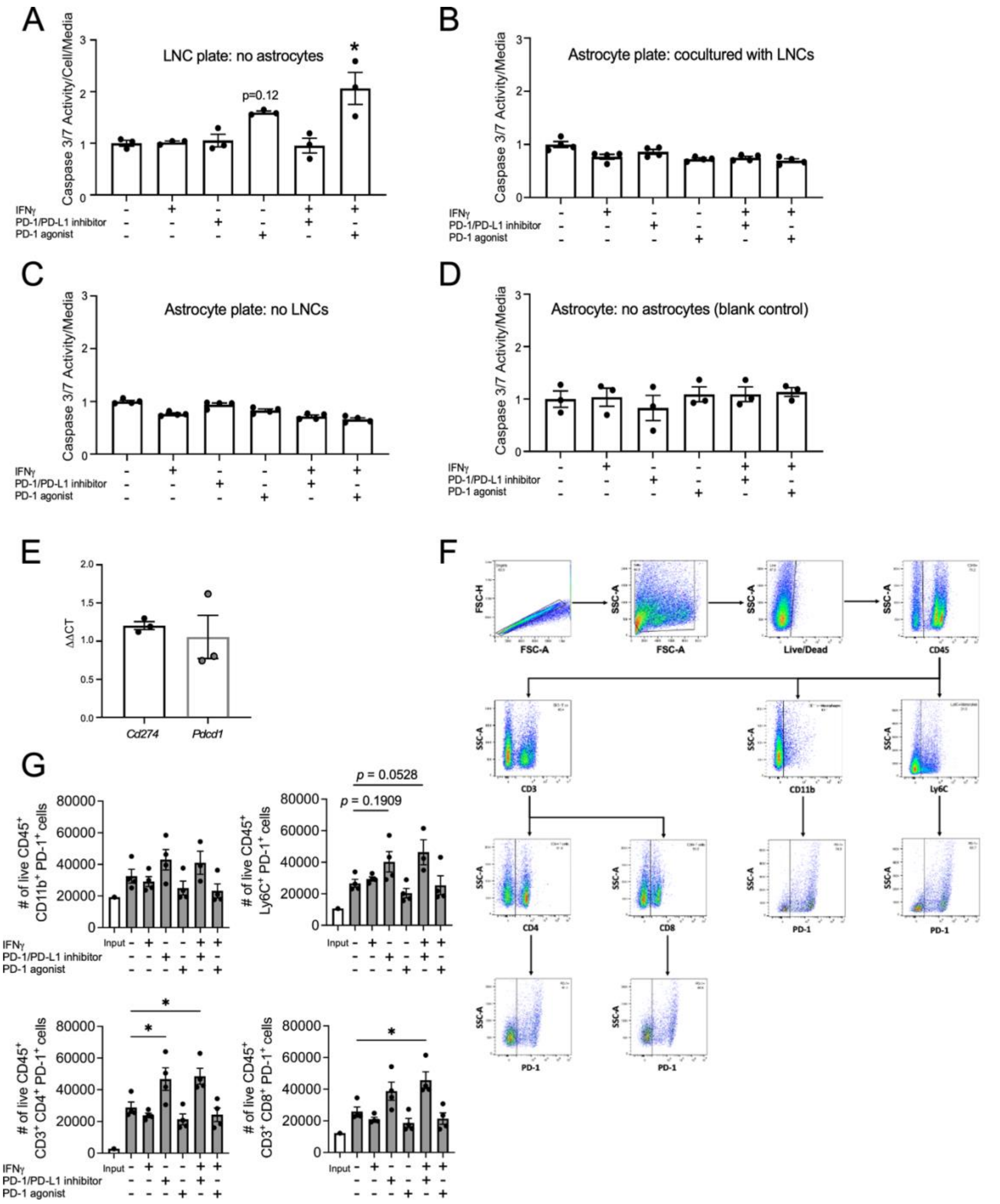

Supplemental Figure 2. PD-1 agonism and PD-1/PD-L1 antagonism primarily impacts LNCs cocultured with astrocytes. Murine astrocytes and LNCs were harvested and co-cultured in the presence of media alone, 10 ng/ml IFNγ, 100 nM PD-1/PD-L1 inhibitor, and/or 1.0 μg/ml PD-1 agonist for 48 h. Caspase 3/7 activity was measured in (**A**) LNCs cultured alone, (**B**) astrocytes

co-cultured with LNCs following LNC removal, (**C**) astrocytes cultured alone, (**D**) and in a LNC cultured plate following LNC removal to serve as a blank/background control. Caspase 3/7 activity was normalized to cell number. (**E**) Primary murine LNCs were stimulated with and without 10 ng/ml IFNγ for 24 h and RNA transcript levels of *Cd274* and *Pdcd1* were assessed. Data are representative of 2 independent experiments with 3-4 technical replicates each. All data represent the mean ± SEM. *$P < 0.05$ by one-way ANOVA. (**F**) Following culture with astrocytes, LNCs were processed for flow cytometry and gated for singlets, total cells, live cells, CD45, individual cell type and then PD-1. All gates were determined using FMO controls. (**G**) PD-1^+ LNC types were quantified following exposure to treated astrocytes. Data points represent the mean of n=4 technical replicates ± SEM. *$P < 0.05$ by one-way ANOVA compared to media-treated samples.

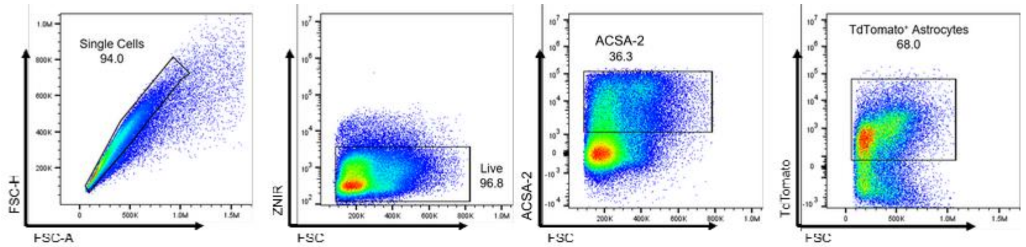

Supplemental Figure 3. Gating strategy to assess recombination efficiency in *Ifngr1*$^{fl/fl}$ *Aldh1l1*-Cre^{ERT2+} mice. The Ai14 reporter strain, which has a *loxP*-flanked STOP cassette, preventing red fluorescent protein (TdTomato) expression, was crossed with *Ifngr1*$^{fl/fl}$ *Aldh1l1*-Cre^{ERT2+} mice. Naïve mice were given tamoxifen i.p. for 5 consecutive days. Spinal cord tissue was then digested and labeled with ACSA-2 to mark astrocytes. Cells were gated for singlets, live cells, ACSA-2 positivity, and then TdTomato positive cells to determine recombination efficiency.

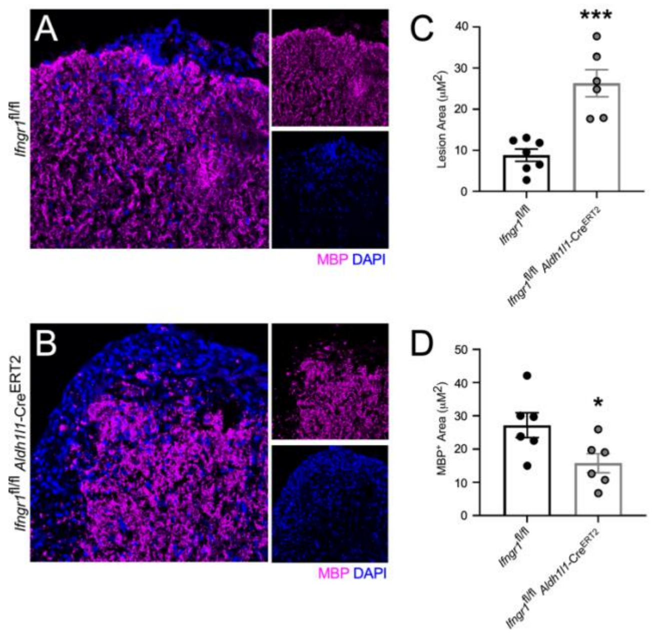

Supplemental Figure 4. EAE lesion characterization in *Ifngr1*$^{fl/fl}$ *Aldh1l1*-Cre^{ERT2+} mice.

EAE was induced in *Ifngr1*$^{fl/fl}$ *Aldh1l1*-Cre^{ERT2+} mice (*n* = 7) and *Ifngr1*$^{fl/fl}$ littermate controls (*n* = 8) and EAE clinical course was blindly monitored. On day 16 ± 1 mice were injected i.p. with tamoxifen for 5 consecutive days to induce recombination. 35 days post-immunization, mice were perfused and the CNS was removed and cryopreserved for IHC analysis. Ventral white matter tracts of the lumbar spinal cord were imaged using confocal microscopy. (**A**) *Ifngr1*$^{fl/fl}$ and (**B**) *Ifngr1*$^{fl/fl}$ *Aldh1l1*-Cre^{ERT2+} tissue sections were labeled for MBP and nuclei were counterstained with DAPI. (**C**) Lesion area and (**D**) MBP positive area were quantified. Data represent the combined mean ± SEM from 2 independent experiments and were analyzed using a two-tailed Student's *t* test. *P < 0.05, ***P < 0.001.

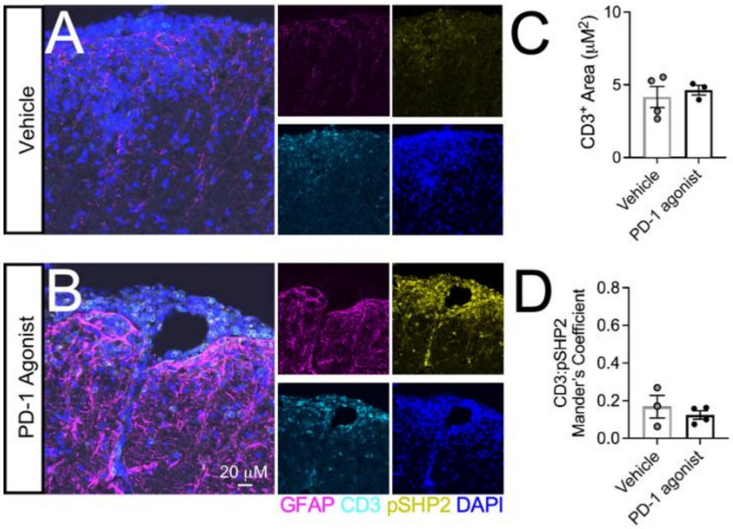

Supplemental Figure 5. PD-1 agonism did not alter T cells during EAE. EAE was induced in WT C57Bl/6J mice. Clinical course was blindly monitored. One day after peak disease, a PD-1 agonist or vehicle control treatment was randomly assigned and injected i.p. for 5 consecutive days. Data are representative of two independent experiments. 25 days post-immunization, mice were sacrificed, and the CNS was collected and cryopreserved for IHC analysis. Ventral white matter tracts of the lumbar spinal cord were imaged using confocal microscopy. Spinal cord tissue from (**A**) vehicle- and (**B**) PD-1 agonist-treated mice were labeled for GFAP, CD3, pSHP2, and nuclei were counterstained with DAPI. (**C**) Total CD3 and (**D**) colocalization of CD3 with pSHP2 were quantified using ImageJ. Data represent the mean ± SEM and were analyzed using a two-tailed Student's *t* test.

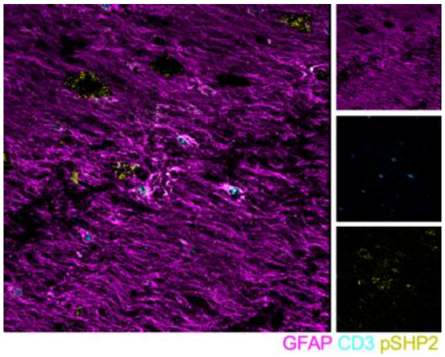

Supplemental Figure 6. T cells at the chronic active lesion rim do not appear to express PD-1. Using human post-mortem MS tissue, chronic active lesions were cryopreserved, sectioned, labeled for GFAP, CD3, and PD-1, and imaged at 20x magnification using confocal microscopy.
